# Prevalence, pathogenesis, therapy, and prevention of cardiovascular events in patients with community-acquired pneumonia

**DOI:** 10.1186/s41479-016-0011-0

**Published:** 2016-07-21

**Authors:** Charles Feldman, Ronald Anderson

**Affiliations:** 1 Division of Pulmonology, Department of Internal Medicine, Charlotte Maxeke Johannesburg Academic Hospital, Johannesburg, South Africa; 2 Faculty of Health Sciences, University of the Witwatersrand, Johannesburg, South Africa; 3 Institute for Cellular and Molecular Medicine, Department of Immunology, Faculty of Health Sciences, University of Pretoria, Pretoria, South Africa; 4 Department of Internal Medicine, University of the Witwatersrand Medical School, 7 York Road, Parktown, 2193 Johannesburg, South Africa

**Keywords:** Macrolides, Platelets, Pneumococcus, Pneumolysin, Statins, Pneumococcal vaccines

## Abstract

It is now well recognised that cardiac events occur relatively commonly in patients with acute community-acquired pneumonia. While these events are more frequent in patients with underlying risk factors—such as those with underlying chronic cardiovascular and respiratory comorbidities, the elderly, and in nursing home residents—they also occur in patients with no underlying risks other than severe pneumonia. Recent research elucidating the underlying pathogenic mechanisms related to these cardiac events has indicated a probable role for platelet activation, which is possibly exacerbated by pneumolysin in the case of pneumococcal infections. This, in turn, has resulted in the identification of possible therapeutic strategies targeting platelet activation, as well as the cardio-toxic activity of pneumolysin. These issues represent the primary focus of the current review.

## Background

For a considerable period of time, investigators have been reporting on the occurrence of cardiac events in patients with acute respiratory tract infections, including community-acquired pneumonia (CAP) [1, 2]. Among these cardiac events are acute coronary syndromes (acute myocardial infarction [AMI]), acute or worsening cardiac arrhythmias and new or worsening congestive heart failure (CHF) [2]. The features of the association between acute coronary events and arrhythmias and CAP appear to fulfill the criteria for causality—namely, that pneumonia is a cause of these events; whereas, in the case of CHF, it appears the cause–effect relationship may be bidirectional [2]. More recent studies are beginning to give insight into the magnitude of this problem, also indicating that these events tend to be particularly common early on during the course of CAP, and also tend to be more common in patients with certain risk factors, including older age, nursing home residence, pre-existing cardiovascular disease, and in more severe pneumonia [2]. Nevertheless, it is important to recognise that in up to 50 % of cases with CAP in which acute cardiac complications occur, there has been no prior history of overt chronic cardiac disease [3].

It is interesting to reflect on the fact that in patients with CAP, 50 % of the mortality within the first month is due to comorbidities and not directly due to the consequences of CAP (e.g. respiratory failure or sepsis), and that cardiovascular diseases are also among the main causes of death following a CAP episode [4, 5]. Furthermore, cardiovascular events (CVEs) are recognised to be the primary causes of clinical failure in patients with CAP and should be actively looked for in patients not responding to standard medical care [6]. Importantly, it is now recognised that acute cardiac events occurring during the course of CAP are associated with negative impacts on both short-term and long-term outcomes [2, 6]. This article will review the clinical studies of cardiac events in patients with CAP, describe the likely pathogenic mechanisms involved in this interaction, and discuss possible therapeutic and preventative strategies to manage these events, particularly in pneumococcal CAP.

## Clinical studies

### Acute infections and the onset of acute coronary syndromes

In 1984, Spodick and colleagues [7] prospectively investigated 150 patients hospitalised with AMI and an equivalent number of age-, sex- and date-of-admission-matched controls. They noted that there was an increased risk (odds ratio [OR] 2.2; *p* < 0.02) of the occurrence of minor acute respiratory symptoms in the patients with AMI compared with the controls, and suggested that further investigations should be undertaken to determine if there was a pathogenic relationship between acute respiratory symptoms, considered likely to be of viral origin, and the onset of AMI. Over many subsequent years, the association between acute infections, most commonly respiratory infections, but also *Staphylococcus aureus* bacteraemia, urinary tract infections and gastroenteritis, and the occurrence of acute coronary syndromes has been documented [1].

### CAP and acute cardiac events

Seedat and colleagues [8] undertook one of the first studies investigating acute cardiac events in hospitalised patients with acute CAP. The authors prospectively studied consecutive patients with CAP who had no prior or underlying cardiorespiratory illnesses. In addition to recording demographic, clinical, microbiological, laboratory, and outcome data, they also performed electrocardiographic (ECG) and echocardiographic investigations and measured serum enzyme, including cardiac enzyme levels (creatinine kinase [CK], CK-MB fraction, and lactate dehydrogenase [LDH]). A number of ECG changes were documented, which returned to normal after a mean of 2 days in survivors. The so-called S1, Q3, T3 pattern on ECG was associated with CK-MB leak, hypoxia and a high Simplified Acute Physiology Score (SAPS). The presence of P pulmonale, right axis deviation and clockwise rotation correlated with hypoxia and high SAPS score. Clockwise rotation also correlated with raised serum LDH and CK-MB levels and high pulmonary artery pressures. The overall patient mortality was 10.8 % and a number of negative prognostic factors were documented. While there was no association between the ECG changes and mortality, raised LDH, CK and CK-MB levels correlated, among other factors, with need for intensive care unit (ICU) admission and mortality.

Ramirez and colleagues [9] undertook a retrospective observational study of 500 consecutive cases with CAP enrolled into the Community-Acquired Pneumonia Organization (CAPO) study. On hospital admission, AMI was documented in 29/500 (5.8 %) patients, including 13/86 (15 %) patients with severe CAP, and AMI was subsequently documented during hospitalisation in 13/65 (20 %) patients who experienced clinical failure. Significant associations between AMI and Pneumonia Severity Index (PSI) (*p* = 0.05) and clinical failure (*p* = 0.04) were noted after risk adjustment. The authors concluded that in patients with CAP experiencing clinical failure, AMI should be considered as a possible aetiology. Other investigators have confirmed that acute cardiac events [10] and both cardiovascular and cerebrovascular events [11] are common and are associated with significant morbidity and mortality in patients with CAP.

Perry et al. [12] examined a cohort of 50,119 subjects extracted from databases of the Department of Veterans Affairs (VA) from the period October 2001 to September 2007. The patients, of whom 98 % were males, had a mean age of 77.5 years (standard deviation 6.7 years), and the 90-day incidence of CVEs was 1.5 % for AMI, 10.2 % for CHF, 9.5 % for arrhythmia, 0.8 % for unstable angina, and 0.2 % for stroke, with most events occurring during hospitalisation. The authors suggested that CVEs may play a significant role in CAP patient mortality and that research was needed to determine whether appropriate interventions could reduce the number of these CVEs.

Corrales-Medina et al. [13] studied 1,343 inpatients and 944 outpatients with CAP for 30 days after their presentation. They noted cardiac events (defined as new or worsening CHF, new or worsening arrhythmia, or AMI) in 358 (26.7 %) of inpatients and 20 (2.1 %) of outpatients. More than half of these events were documented in the first 24 h, and the factors that were particularly associated with their diagnosis included older age, nursing home residence, pre-existing cardiovascular conditions and indicators of pneumonia severity. These authors also considered that further studies on risk stratification of CAP patients for these complications and strategies for their prevention and treatment need to be undertaken.

### Risk stratification for acute cardiac events/cardiac complications in patients with CAP

Viasus and colleagues [3] undertook a study to investigate risk stratification for these acute cardiac events and their prognosis. This was a prospective observational study of a cohort of 3,921 hospitalised adults with CAP, in whom a logistic regression analysis was performed to document predictors for these acute cardiac events and their mortality. Overall 315 (8 %) of these patients had ≥1 acute events during hospitalisation and multivariate analysis documented a number of risk factors for these events, which were used to develop a prognostication rule that had an area under the receiver operating characteristic curve of 0.73 (95 % confidence interval [CI] 0.70–0.76). The case fatality rate was significantly higher in patients who had an acute cardiac event (19.4 %) than in patients who did not have such an event (6.4 %; *p* < 0.001). The authors concluded that such a rule could be used to identify CAP patients at high risk of cardiac complications.

Other investigators have sought to develop clinical rules for risk stratification in patients hospitalised with CAP [14]. In this study, 2 cohorts of patients were selected, which represented the derivation and validation cohorts. A point score system was developed from the derivation cohort which, when applied to the validation cohort, had good discrimination and outperformed the PSI in predicting cardiac complications.

Aliberti and colleagues [15] studied the prevalence, clinical characteristics, risk factors and impact on outcome of AMI *versus* other CVEs in patients with CAP. They documented that hospital mortality was higher in patients with AMI in comparison to those who had other CVEs and suggested that this should be considered when planning interventional studies.

### Impact of prior therapy on risk of cardiac events in patients with CAP

Using the Department of VA administrative database, Soto-Gomez et al. [16] studied hospitalised CAP cases ≥65 years, in which the patients received antibiotics within 48 h and had no prior diagnosis of a cardiac arrhythmia. They documented that 3,919/32,689 (12 %) patients developed cardiac arrhythmia within 90 days of admission. Increased age, history of CHF, and need for mechanical ventilation or vasopressors were significantly associated with the occurrence of an arrhythmia and, even after adjustment for confounders, incident cardiac arrhythmias were associated with increased 30-day and 90-day mortality. However, beta-blocker use was associated with a decreased risk of arrhythmia (OR 0.92 [95 % CI 0.85–0.99]). Other investigators documented that 376/3,068 (12 %) hospitalised patients with CAP had a CVE and, while hyperlipidaemia, more severe pneumonia and *S. aureus* and *Klebsiella pneumoniae* pneumonias were associated with an increased risk, statin use was associated with a decreased risk [17]. Both these investigators suggested that studies of cardioprotective therapies in patients with CAP should be undertaken to determine if they are able to attenuate cardiac risks [16, 17].

### Long-term cardiovascular complications of CAP

More recently, a number of investigators have studied long-term cardiovascular complications of CAP [18–21]. In one investigation adult patients with CAP initially hospitalised in a tertiary hospital were followed up for 1 year, post-discharge [18]. Overall, 93/1,284 (7.2 %) patients died within 1 year of hospital discharge (68 [73.1 %] in the first 6 months). The main reasons for mortality were infectious diseases, mainly pneumonia, and acute CVEs. A number of risk factors were noted, including chronic obstructive pulmonary disease, diabetes, cancer, and dementia, which could help identify cases at high risk of long-term mortality. Another study documented cardiac complications (sometimes more than one) in 55/310 (18 %) hospitalised CAP patients, which included AMI or a new episode of atrial fibrillation in 32 and 30 patients, respectively [19]. However, during the follow-up, 89 patients died (51 % of whom had experienced an in-hospital cardiac complication, *versus* 26 % who had not; *p* < 0.001); in addition, 73 had a CVE (47 % of patients who had experienced an in-hospital cardiac event, *versus* 19 % who had not; *p* < 0.001). Among other factors, in-hospital cardiac events were significantly associated with both overall mortality and CVEs during follow-up. A third study in elderly patients with CAP (≥65 years) [20] documented the occurrence of new onset of heart failure in both the intermediate and long-term (even after 5 years) following hospitalisation, which had not been present on hospital admission. These same authors confirmed in an additional study [21] that hospitalisation for CAP is associated with both short-term and long-term risk of cardiovascular disease and while the risk of cardiovascular disease was greatest in the first year following hospitalisation, it remained higher than in controls for up to 10 years of follow-up.

Currently, a study is being undertaken to document whether various diverse inflammatory and immune response mediators may be used as markers of both short-term and long-term (1 year) occurrence of cardiovascular disease and mortality [22].

### Non-cardiac complications in patients with AMI

Other investigators, in contrast, have studied patients with AMI and looked for the occurrence of acute, life-threatening and/or significant non-cardiac events that were present on hospital admission of these patients [23]. Such events occurred in approximately one-third of patients with AMI. Of the 1,145 patients studied, pneumonia was noted in 7.2 % (*n* = 82), which were classified as life-threatening in 1.5 % (*n* = 17) and significant in 5.7 % (*n* = 65). This study was undertaken early on in 2006 and, given what we have subsequently learned about the occurrence of cardiac events in patients with pneumonia, it would be interesting to speculate whether the primary event in at least some of these cases with AMI and severe pneumonia may have been the pneumonia itself.

### Pneumococcal pneumonia and acute cardiac events

In 2007, Musher and colleagues [24] published the first study investigating the possible association between pneumococcal pneumonia and acute cardiac events. This was a study undertaken over a 5-year period (2001–2005) aimed at identifying hospitalised patients with pneumococcal pneumonia who developed AMI, significant arrhythmia (namely atrial fibrillation or ventricular tachycardia), or new onset or worsening of CHF. Overall, 33/170 (19.4 %) patients had one or more of these cardiac events: 12 had AMI (2 also had arrhythmia and 5 had new or worsening CHF), 8 had an arrhythmia (6 also had new onset CHF) and 13 had new or worsening CHF. There was a significantly higher mortality in those patients with pneumococcal pneumonia who had an acute cardiac event compared with those that did not (*p* < 0.008).

### Cardiac complication in patients with CAP: systematic review and meta-analysis

Corrales-Medina et al. [25] undertook a systematic review and meta-analysis to document the incidence of major cardiac complications within 30 days in patients diagnosed with CAP in order to determine the magnitude of the problem. Among the 17 articles (68 %) that had reported on in-patient cohorts of CAP, data extracted from appropriate studies indicated that incidence rate of overall cardiac complications was 17.7 %, heart failure was 14.1 % (range 7–33 %), acute coronary syndromes was 5.3 % (range 1–11 %) and cardiac arrhythmias was 4.7 % (range 1-–11 %). Major conclusions from this analysis were that cardiac complications were common in patients with CAP and needed to be better appreciated by physicians and patients alike, with early recognition and appropriate management. Furthermore, the investigators indicated the need for a better understanding of pathogenic mechanisms, of risk factors, and of the impact of these cardiac events on patient outcome. In addition, as indicated by these and other investigators, there needs to be a better understanding of interventions to treat these complications (e.g. with the use of cardioprotective medications), as well as to prevent them (e.g. with use of influenza virus and pneumococcal vaccination) [26]. Possible therapeutic and preventative strategies, preceded by a brief overview of current concepts in pathogenesis including the role of pneumolysin in CAP-associated CVEs, are discussed in more detail below.

## Pathogenic mechanisms of CAP-associated CVEs

### The role of platelets

Mirsaeidi et al. [27], recognising that platelets were inflammatory cells that played an important role in host antimicrobial defences, undertook a study to evaluate the association between abnormalities of the platelet count and 30-day mortality in patients with CAP. These investigators documented that the occurrence of either thrombocytopaenia or thrombocytosis were associated with in-hospital mortality in patients with CAP and that these abnormalities of the platelet count were better predictors of outcome than similar abnormalities of the leukocyte count.

Recent and novel insights into the pathogenesis of CAP-associated CVEs have been provided by the findings reported in a study by Cangemi et al. [28]. This study, which did not distinguish between viral and bacterial CAP, confirmed the high risk for development of myocardial infarction, which peaked within 2 days of hospitalisation. Notwithstanding disease severity and heart ejection fraction, significant predictors of myocardial infarction included elevated concentrations of the circulating biomarkers of platelet activation, soluble CD40 ligand, P-selectin (CD62P) and thromboxane B2 (a surrogate for thromboxane A2), as well as the mean platelet volume [28]. The contention that platelet activation plays a pivotal role in orchestrating inflammatory events which trigger, or more likely exacerbate, vascular damage and dysfunction is supported by the steadily increasing recognition of the critical role played by these cells in mediating inflammatory and immune responses [29–33].

Pro-inflammatory mechanisms by which platelet activation may contribute to the pathogenesis of bacterial CAP in particular have recently been reviewed elsewhere [30] and are briefly considered and updated here. The most significant of these is activation and aggregation of platelets by bacteria and their products such as cell-wall components (lipoteichoic acids/peptidoglycans/endotoxins), DNA, and toxins such as pneumolysin and staphylococcal α-haemolysin [34, 35]. These, in turn, interact with and activate Toll-like receptors (TLRs) and other types of pathogen-recognition receptors that are expressed on platelets [29, 30, 36]. In this context, it is, however, noteworthy that a role for platelet TLRs in sepsis-mediated endothelial dysfunction has been challenged based on data derived from an experimental model of Gram-negative sepsis [37].

Bacteria, specifically those belonging to the *Staphylococcus* and *Streptococcus* genera, have also been reported to mediate platelet activation by a complex and unusual mechanism involving the interaction of these microorganisms with platelet factor 4 (PF-4) present in platelet α-granules [38]. The resultant complex binds to immunoglobulin G FcRIIA receptors, resulting in activation of the platelet pro-thrombotic, fibrinogen-binding integrin, GPIIb/IIIa [38].

In addition to the aforementioned mechanisms, we have recently observed that the major protein virulence factor of the pneumococcus, pneumolysin, activates the production of platelet-activating factor (PAF) by human neutrophils in vitro. These effects, which were observed at pathologically relevant concentrations of the toxin, were dependent on its cholesterol-binding, pore-forming activity and unrelated to interaction with TLR4 [39]. In this context, it is noteworthy that PAF has been reported to contribute significantly to pneumolysin-induced lung injury in a murine model of experimental infection [40]. We have also observed that pneumolysin, also at pathologically relevant concentrations, activates platelets directly, according to upregulated surface expression of the adhesion molecule CD62P, a constituent of platelet α-granules. This pro-adhesive mechanism, which favours the binding of platelets to both neutrophils and vascular endothelium, was also dependent on the pore-forming activity of pneumolysin [41].

These various mechanisms of platelet activation not only favour homotypic aggregation resulting in the formation of platelet clusters, but also heterotypic aggregation of these cells with neutrophils and vascular endothelium. In this setting, activated platelets act as bridges promoting neutrophil clusters, as well as binding of neutrophils to vascular endothelium mediated by CD62P and other pro-adhesive mechanisms [30, 32, 42]. If poorly controlled, systemic inflammatory mechanisms involving platelets, neutrophils and vascular endothelium predispose to endothelial damage and dysfunction, promoting microvascular damage and coagulation [31].

Potential mechanisms of endothelial damage, resulting in exposure of sub-endothelial collagen and von Willebrand factor, include exposure to cytotoxic neutrophil-derived reactive oxygen species and proteases. In addition, activated platelets have been reported to induce the formation of neutrophil extracellular traps (NETs) at sites of plaque rupture in vivo [43]. NETs contribute to atherothrombosis by several mechanisms. These include the cytotoxic actions of their histone constituents on endothelium, as well as expression of functional, neutrophil-derived tissue factor [44].

### Effects of pneumolysin

Pneumolysin is increasingly implicated in the pathogenesis of life-threatening acute cardiac complications in CAP, albeit based on data derived from experimental animal models. Brown et al. [45] have reported that experimental invasive pneumococcal disease (IPD) in mice and rhesus macaques was associated with myocardial damage and formation of cardiac microlesions. Translocation of the pneumococcus into the myocardium appeared to be dependent on the pneumococcal adhesins, choline-binding protein A and phosphorylcholine, while microlesion formation was mediated by pneumolysin [45]. Similar microlesions were detected in cardiac sections from patients with fatal IPD [45]. The authors concluded “that the microlesions and the scarring that occurs thereafter may explain why adverse cardiac events occur during and following IPD” [45].

Essentially similar findings have been reported by Alhamdi et al. [46]. The authors, who also used a murine model of IPD, observed that infection with pneumolysin-expressing pneumococci, but not with strains deficient in the toxin, resulted in significant elevations in circulating cardiac troponins and myocardial injury [46]. These injurious effects on the myocardium were mimicked by intravenous administration of recombinant pneumolysin, but not by a mutant toxin devoid of pore-forming activity, and were attenuated by co-administration of pneumolysin-sequestering liposomes [46]. Mechanistically, exposure of murine cardiomyocytes to sublytic concentrations of the toxin resulted in intense calcium influx and overload followed by membrane depolarisation [46], as previously reported for human neutrophils [47]. These disruptive effects of the toxin on cardiomyocytes were associated with a “progressive reduction in intracellular calcium transient amplitude, a key determinant of contractile force” [46].

Given its dual mechanism of mediation of cardiac dysfunction—firstly, direct damage to cardiomyocytes; and secondly, pro-inflammatory, pro-thrombotic actions involving activation of platelets and neutrophils and damage to vascular endothelium—pneumolysin appears to be a priority target in the prevention of CAP-associated CVEs.

## Treatment and/or prevention of CAP-associated CVEs

Three potential strategies will be considered here: (i) adjunctive anti-platelet pharmacological agents; (ii) strategies targeting pneumolysin; and (iii) pneumococcal immunisation.

### Anti-platelet strategies

These have been covered extensively in recent reviews [31, 48] and will be considered briefly here. Agents which target platelet activation fall into several categories, targeting both mediators of activation and their receptors, and include, but are not limited to, the following:

- inhibitors of production of thromboxane A_2_
- antagonists of thromboxane A_2_ receptors
- antagonists of ADP-activated P2Y12 receptors
- antagonists of thrombin-activated proteinase-activated receptor 1
- GPIIb/IIIa inhibitors.

Given the relatively recent appreciation of the role of platelet activation in the pathogenesis of CAP-associated CVEs, it is understandable that no compelling supportive data on the preventive potential of anti-platelet therapies in this setting currently exists. However, of the various categories of anti-thrombotic agents, aspirin, which decreases the production of thromboxane A_2_ via its inhibitory effects on cyclooxygenase-1, represents a relatively safe and inexpensive option in the setting of prevention of CAP-associated CVEs.

In this context, it is noteworthy that the study alluded to earlier by Cangemi et al. [28] describing the association between CAP, platelet activation and CVEs, failed to detect a protective effect of aspirin administered at a daily dose of 100 mg on the occurrence of myocardial infarction. The authors attributed the lack of benefit to an inadequate dose of aspirin. In a subsequent prospective study in which a larger number (*n* = 1,005) of elderly hospitalised patients with CAP was recruited, these same authors investigated the effect of administration of aspirin (100 mg daily before and during hospital admission) on the 30-day mortality rate and overall incidence of non-fatal CVEs [49]. During the follow-up period, 16.2 % of patients died: 19/390 (4.9 %) in the group of aspirin-users and 144/615 (23.4 %; *p* < 0.001) in the group of non-users, with a hazard ratio for total mortality of 2.07 (*p* = 0.029) [49]. Non-fatal CVEs occurred in 7 % of patients, 4.9 % and 8.3 % of whom were aspirin-users and non-users, respectively (OR 1.77; *p* = 0.04).

Other studies, one focused on CAP [50], others on acute lung injury (ALI)/acute respiratory distress syndrome (ARDS) [51–53] have reported significant benefit of pre-hospitalisation administration of anti-platelet therapies, including aspirin, on various outcomes such as requirement for intensive care treatment, length of hospital stay, incidence of ALI/ARDS, and mortality. These findings were not, however, confirmed in a large multicentre, international observational study (*n* = 3,855 patients) focused on the potential benefit of pre-hospitalisation administration of aspirin on development of ALI following hospital admission [54]. Although univariate analysis revealed a reduced incidence of ALI in patients receiving aspirin therapy (OR 0.65; *p* = 0.010), this protective effect was attenuated using propensity score analysis (OR 0.70; *p* = 0.072) [54]. Nonetheless, the authors conceded that prospective intervention trials were justified. In this context, a recently published study [55], albeit involving a relatively small number of patients with ARDS (*n* = 202) admitted to a single medical and surgical ICU over an 18-month period, is noteworthy. The authors observed that administration of aspirin pre-hospitalisation and during ICU stay was associated with a significant reduction in mortality (OR 0.38; *p* = 0.04) [55].

Although aspirin in particular, and possibly other anti-platelet therapies show promise in the prevention of the major cardiovascular and pulmonary complications of CAP, there is a clear need for stringently controlled, definitive intervention trials in this setting.

### Strategies targeting pneumolysin

Given the well-recognised involvement of pneumolysin in the pathogenesis of pneumococcal CAP and its major pulmonary complications [40, 56, 57], together with increasing evidence implicating the toxin in the aetiology of CVEs associated with this condition, pneumolysin is clearly a priority target for therapeutic intervention. Two types of strategy exist: firstly, the use of agents, specifically macrolide and macrolide-like antibiotics, which target production of pneumolysin by the pneumococcus; and secondly, agents which attenuate the cytotoxic and pro-inflammatory activities of the toxin, specifically statins and intravenous cholesterol-risk liposomes.

#### Macrolides

Unlike bactericidal antibiotics, such as beta-lactams, which potentiate the release of pneumolysin [58], macrolides, via their predominantly bacteriostatic inhibitory effects on protein synthesis, potently suppress the production of the toxin by the pneumococcus. These inhibitory effects of macrolides on the production of pneumolysin, which are evident at sub-inhibitory concentrations of these agents [59, 60] even in the setting of macrolide resistance [61], may underpin their utility as adjuncts to beta-lactams in the treatment of severe pneumococcal disease [58, 62].

#### Statins

Statins, almost exclusively in the setting of pre-hospitalisation administration, have been reported in a number of studies to reduce CAP-related mortality and to protect against the development of CAP [reviewed in 31]. In the case of pneumococcal CAP, statins, specifically simvastatin, have been reported to attenuate the harmful activities of pneumolysin. Apart from protecting isolated human brain microvascular endothelial cells [63] and airway epithelial cells [64] against the cytotoxic actions of pneumolysin, simvastatin has also been found to reduce mortality in a murine sickle-cell disease model of experimental pneumococcal infection [63]. While these activities of simvastatin appear to be related to interference with pneumolysin/membrane cholesterol interactions, it is also noteworthy that statins possess alternative anti-inflammatory activity unrelated to their membrane cholesterol-lowering activities that target various inflammatory cell types, including platelets [31].

However, as with conventional anti-platelet agents, the potential of statins to target CVEs, as well as pulmonary complications, in patients with recently diagnosed CAP awaits the outcome of stringently controlled intervention trials.

#### Cholesterol-enriched liposomes

Intravenous co-administration of penicillin together with liposomes containing high concentrations of cholesterol in particular, as well as sphingomyelin, within 10 h after experimental infection of mice, has been found to protect against development of septicaemia and mortality in comparison with control animals or those treated with penicillin only [65]. The authors attributed the protective effects of the liposomes to sequestration of pneumolysin. Although innovative, the practicality of this approach in the clinical setting is debatable.

### Pneumococcal immunisation

The protective effect of immunisation in the prevention of influenza-associated acute coronary events is well recognised [66–68], suggesting that a similar relationship may exist in the case of pneumococcal polysaccharide vaccine 23 (PPV23), which has been available since 1983 [69]. In this context, it is noteworthy that a large case-control study documented a significant decrease of >50 % in the rate of myocardial infarction in recipients of PPV23, which was evident from 2 years and onwards following administration of the vaccine [70]. More recently, a systematic review and meta-analysis of 11 cohort studies covering the period 2000–2013, which included 332,267 participants, concluded that administration of PPV23 conferred transient protection against both myocardial infarction and cerebrovascular events [71].

These apparently protective effects of PPV23 in the prevention of CVEs have, however, been challenged by the findings of a number of large case-control and population-based cohort studies which failed to confirm protection against AMI, ischaemic stroke, transient ischaemic events and all-cause mortality [66, 67, 72, 73]. In another population-based cohort study of adults presenting with CAP, PPV23 immunisation was associated with a 58 % reduction in acute coronary syndrome events occurring within 90 days of pneumonia [73]. However, sensitivity analyses revealed that the protective effect of PPV23 was most likely attributable to a confounding “healthy vaccinee” effect [74].

Issues which should be considered in future studies designed to evaluate the potential of pneumococcal immunisation in protecting against the development of CVEs, either in the general population or those with CAP include: (i) the confounding potential of prior influenza immunisation, which may result in overestimation of the benefit of pneumococcal immunisation [75]; and (ii) assessment of the benefit of new generation pneumococcal vaccines, specifically pneumococcal conjugate vaccine 13 (PCV13) with improved immunogenicity. While a recent study [76] has documented significant protection against pneumococcal infection in the elderly with the use of PCV13, there have been no studies specifically addressing the impact of PCV13 immunisation on CVEs. However, given the increasing realisation of the probable involvement of pneumolysin in the pathogenesis of CVEs secondary to pneumococcal CAP, the inclusion of an immunogenic pneumolysoid with PCV13 (or a similar polysaccharide conjugate vaccine) may offer maximal benefit [77].

Potential therapeutic and preventative strategies are shown in Table 1.

**Table 1:** Potential therapeutic/preventative strategies targeting cardiac events in patients with community-acquired pneumonia

• Anti-platelet agents	
o inhibitors of production of thromboxane A_2_	
o antagonists of thromboxane A_2_ receptors	
o antagonists of ADP-activated P2Y12 receptors	
o antagonists of thrombin-activated proteinase-activated receptor 1	
o GPIIb/IIIa inhibitors	
• Agents targeting pneumolysin	
o macrolides	
o statins	
o cholesterol-rich liposomes	
• Immunisation	
o PCV 13 with or without immunogenic pneumolysoid	

## Conclusion

The role of CAP in triggering often fatal acute coronary syndromes, almost exclusively in hospitalised patients, is clearly well recognised. The risk for development of cardiac complications is highest soon after diagnosis with about 90 % of these events detected within 7 days and more than half within the first 24 h. Mortality attributable to CAP-related cardiovascular abnormalities may be associated with pre-existing plaque formation (AMI, predominantly silent) or to unrelated causes (arrhythmias), the former being more common. The major risk factors include older age, nursing home residence, severity of CAP, chronic cardiovascular and respiratory comorbidities and smoking. Although most evident in the acute phase of CAP, it is, however, noteworthy that the risk for development of new-onset heart failure in elderly adults previously hospitalised for CAP extends to 1 year, albeit at a lower rate, remaining evident up to 10 years after initial diagnosis. This includes patients who had previously experienced and survived intra-hospital CAP-related cardiac complications. In this context, CVEs have been reported to account for more than 30 % of deaths at long-term follow-up averaging 1–5.4 years, possibly due to CAP-related exacerbation, as well as possibly persistence of the inflammatory mechanisms described above. Promising therapeutic strategies include those targeting platelet activation, especially aspirin. In the case of pneumococcal CAP, macrolides, statins and cholesterol-loaded liposomes interfere with the production or bioactivity of pneumolysin, while new generation vaccines offer the prospect of prevention.

### Search strategy

The authors searched PubMed (Medline) and Google using the following combinations of topics: CAP and/or community-acquired pneumonia and/or pneumonia AND acute cardiac events and/or acute myocardial infarction and/or arrhythmia and/or cardiac and/or cardiac events and/or cardiac failure and/or cardiovascular and/or cardiovascular events and/or congestive cardiac failure and/or congestive heart failure and/or heart failure and/or neutrophils and/or neutrophil extracellular traps and/or pneumococcus and/or pneumolysin and/or platelets and/or platelet activating factor.

## Abbreviations

ALI, acute lung injury; AMI, acute myocardial infarction; ARDS, acute respiratory distress syndrome; CAP, community-acquired pneumonia; CHF, congestive heart failure; CK, creatinine kinase; CVEs, cardiovascular events; ECG, electrocardiographic; ICU, intensive care unit; IPD, invasive pneumococcal disease; LDH, lactate dehydrogenase; NETs, neutrophil extracellular traps; PCV13, pneumococcal conjugate vaccine 13; PPV23, pneumococcal polysaccharide vaccine 23; TLR, toll-like receptor; VA, Veterans Affairs
